# The Institutional Library

**Published:** 1917-04-07

**Authors:** 


### THE INSTITUTIONAL LIBRARY.

### The Effect of Printing on Literature.

Progress, it has often been pointed out, means to many people improvement in the machinery of existence, but that, within the narrow range of history, the word is inapplicable to man's mental or spiritual nature can be shown in many ways, among which philosophy and art provide two excellent examples. It is clear, for instance, that the publication of Plato's works over two thousand years ago is still obviously premature if the understanding which produced them be measured by the intelligence of the average modern voter. Again, in art we note two facts of kindred interest. The first is that the limit of perfection has been reached again and again: there will never be a better sculptor than Pheidias or Rodin, however much the modern or the ancient master may be preferred by private judgment. The second fact is that the instruments of art are the same in all centuries: the chisel has not been superseded; the brush is not out of date; the pen is still the author's tool. The reason is that the practice of any art depends upon a technique of handicraft, and that differences in result and merit depend upon the personal touch by which similar tools are guided in the hands of different people. But the effect of mechanical improvements is often unfortunate in that the change, which is always from the difficult to the easy, from quality to quantity, has the effect of degrading the average level in ways very often overlooked. Let us confine ourselves to one example, the effect of printing on literature.

When you find that the directing personality is separated from the execution, the artist from the craftsman, specialisation of function has set in, and, in art, the result is always decay. It is generally believed that the decline in architecture has been largely due to the fact that modern masons are builders merely, and the modern architect a draughtsman merely, instead of being a master mason who learnt the elements of his art as a craftsman apprentice in the builder's yard. Now the art of letters is that in which the separation between conception and execution is most complete. Literature, as an art, has split into two, the art of composing and the craft of printing. The separation may be even wider yet. The hand is often replaced by the voice which dictates the words, and a man may "write a book" without putting pen to paper. This separation has an evil effect both upon author and compositor. It isolates the author in his study, and confines the compositor to his case. The one has no craft to keep his fingers busy, and thereby to keep his mind sane (he does not take even handwriting seriously or regard bad handwriting as a scamped piece of work), the printer has none of the exhilaration of thinking at his work (he is merely concentrated on the mechanical part of it), and even in casual reading can think of little more than the stops, commas, and literal mistakes in the printed word before him. Literature, alone of the arts to-day, has no handicraft necessary for its production, and the results on authorship are worth considering.

The first is the decay of handwriting: for a modern author writes not to be read, but to be printed. The idea of associating beautiful penmanship with beautiful literature would seem to many paradoxical, and yet the modern indifference to clearness, I will say beauty, of handwriting has blinded people's eyes to the very form of print. Having never cared to study more than the general shape of a letter, how should they observe that subtler thing, its form? To the invention of printing we owe the decay of our handwriting and the vulgarity of our typography. Yet, as anyone who has seen a fine handwriting, Michael Angelo's, for example, cannot but admit, handwriting should be a normal and positive extension of personal beauty.

### The Keeper of the Author's Conscience.

The next result of the invention of printing, whereby the chasm between the art and handicraft of literature was first made, has been, by making authorship easier, to make authors lazy and careless. Any persons who have spent some time in a printing office realise that if most manuscripts were printed as they are received they would be absolutely unintelligible. So careless is the average person that he can hardly write two sentences without a mistake in grammar, and even in sense, while as to spelling and punctuation he "leaves all that to the printer." Many writers cannot even correct their own proofs. A special functionary, therefore, has been created, called the reader, whose duty is to correct mis-spelling; to supply punctuation; to draw attention to the lapses of grammar, and even to remind writers of points of style.

Now it is surely clear that no man can leave an important part of his work to another without that work suffering, and it cannot be denied that a loose manner of writing must encourage looseness of thought. The reader keeps the author's conscience, and no man should keep his conscience but himself.

But the decay of handwriting and looseness of thought consequent upon divided responsibility are not the only evils which literature has suffered through the invention of printing. At least, stated in that bare way, they do not represent the whole loss. It is doubtful if it can be measured in a few words. Perhaps a dogmatic statement of belief and a single illustration may help to indicate its extent. First, then, it seems to the writer that man can only move away from contact with nature, with the actual soil, at his own peril. Men in towns are more hysterical than their country cousins. An industrial civilisation is more unstable than an agricultural one, just as a yokel is more healthy than a City clerk. If the Russian revolution proves successful, and the country is able to carry on the war, it will be because the revolution, as always the work of the towns, is steadied by the great mass of the rural peasantry; for the population of Russia is mainly agricultural. France recovered more quickly from her defeat in 1870 than Germany did from its victory for the same reason. And what is true of country life and agricultural nations is no less true of work which has a handicraft and work which is all done in the head. William Morris, who did both, affirmed that there was something to be gained in clear thinking from the use of one's fingers, and that the man who did not use his fingers lovingly at his work was somehow likely to lose his poise when he took up his pen or made a speech. It may be difficult to prove this, but it is a fact. The earth to which we return makes even in life a claim upon us. In life, as in death, we must run to her for rest. Now for the illustration.

It was the present writer's fortune at one time to read through a mass of writing which had been dictated (not written) by a distinguished man of undoubted ability. The "writing" often seemed so loose, turgid, and incoherent that one could not help wondering how its author's reputation had been gained. On one occasion, when alone on his travels, he was compelled to write an important article with his own hand and pen. The difference in quality was astonishing. It was clear, terse, and arresting to read. More time had been spent on every word of it, since the pen moves more slowly than the mind. Could it be, one asked oneself, that the reputation had been made when the man was too poor to afford a secretary, and had been obliged to do his own penmanship himself?

It should be added, in conclusion, that the tonic effect of manual work on the mind was personally brought home to the present writer when, as a boy, he used to spend his holidays on walking tours. That slow rhythmic exercise in the open air was found to clarify his mind. Wordsworth and all the nature poets have noticed it. Indeed, to sweat seems necessary to virtue; and later the lesson was reinforced when a country house enabled him to indulge in gardening, and has been clinched once and for all by his recent experience in digging the ground for potatoes! While turning the sods, he thought of William Morris's words, of the curious fact that the author is the only artist who has no handicraft to practise, and of the explanation which this suggests concerning the contempt in which the art of writing is held. The question then arose as to the relation of handwriting to literature, and of how far the decay of handwriting had been the cause as well as the accompaniment of the ugliness of modern typography, and of the atrophy (for it amounts to that) of our sense for beauty of form in the design of single letters. If authors wrote to be read, instead of merely to be printed, would they not write more clearly in a double sense? How much have we not lost through the invention of printing? The writer submits that this question will bear more meditation than he can expect most readers to be willing or able to give to it.

### An Official Document to be Studied.

### The Second Annual Report of the Board of Control.

For the year 1915.

This report, which is for the year 1915, was ordered to be printed only on February 15, 1917, so much has the war interfered with the routine work of Government Departments. But for the war, the Board of Control, which has superseded the old Lunacy Commission, would by this time have got into its stride, and we should have had a great deal of very interesting matter concerning the working of the Mental Deficiency Act. As it is, that Act, though it is nominally in force, is scarcely in operation. It requires a great expenditure, which will now and for many years to come be very difficult, and the energies of the Board of Control have been drafted off in other directions, for no fewer than ten of the large county and borough asylums have been turned into war hospitals, and the work of preparing and organising them, and of redistributing into other asylums the patients they contained, has fallen under the direction of the Board of Control. The old Lunacy Commission had but six working Commissioners. The Board of Control has no fewer than ten, and in addition is assisted by three inspectors, officers unknown to the old Commission; but yet the new Commissioners are already as much overworked as the old, and have already had to depart from the strict requirements of the law with regard to visitation.

In spite of all this difficulty, the report of the new body is much more interesting than those of the old Commissioners used to be. For one thing, it refers to more stirring times, but for another it is more vivacious, more fully alive, less a document of routine and less a mere repetition, *mutatis mutandis*, of previous reports. In order to release as many medical men as possible for active service, the Board has consented to forgo many of the entries and returns and statistical items that figure so largely and so unnecessarily in asylum reports, and make so large a proportion of the Blue-book itself. A very large proportion of the time of asylum medical officers is taken up with this routine work, the great bulk of which is of no use to any human being, except to provide work for the compositors who are employed to set it up; and having regard to the reverence and admiration that all Government Departments have for statistics, it is much to the credit of the Board of Control that it should have made this wrench, and consented to do without what it loves so dearly.

There is much in this report that is interesting and that we would willingly comment on at length if space allowed. One matter, at any rate, must be mentioned. The Board draws attention to the large number of imbecile or feeble-minded women of child-bearing age, unmarried, and with numerous illegitimate children. It was the existence of this class of women that provided one of the chief motives, if not the chief motive, for the passing of the Mental Deficiency Act; yet there they are, breeding like rabbits, going into the workhouse for each confinement, and coming out again when it suits them. In twelve workhouses in one part of England there were found forty-two mentally defective women in urgent need of control. Twenty-three of these women had between them at least fifty-one children, and two were pregnant at the time of the visitation. In one workhouse alone were four feeble-minded women having

**THE INSTITUTIONAL LIBRARY** *(continued)*.

between them sixteen children, all illegitimate, and a prospect of more. All these children and their mothers are, of course, a burden on the rates and on society, and the Mental Deficiency Act, which was passed to put an end to this mode of increasing the population, remains, as far as these people are concerned, a dead-letter. This is a disastrous state of things, and should be a warning to those simple-minded—we do not say feeble-minded—enthusiasts whose remedy for every ill is an Act of Parliament. Here the Act of Parliament has been obtained, and how much the better are we for it?

**The Flogging Craze.** By Henry S. Salt. (London: For the Humanitarian League, by G. Allan and Unwin. Crown 8vo. Pp. 150. Price 2s. 6d. net.)

The science of punishment, Penology as it is called, has advanced very little since the time of Beccaria and Bentham, and this book does not advance it by a hair's breadth. A really philosophic, calm, well-reasoned treatise on punishment would be a very valuable book, but a long and loud scream of indignation, such as is emitted by Mr. Henry S. Salt, does not advance matters, and leaves us no wiser. We are familiar with the sentiments and arguments employed, and we are but too familiar with the epithets, "stupid," "brutal," "cruel," "inept," "disgraceful," "vindictive," "useless," "mischievous," "scandalous," and all the rest of them. We hold no brief for the practice of flogging, and are rather opposed to it than otherwise, but if anything could induce us to advocate it, it would be such an intemperate book as this. If only it displayed more reason and less passion it would serve far better the cause the writer has at heart. It is vain to abuse flogging on the ground that it is torture. All punishment is torture. That is the nature of punishment. Mr. Salt, it appears, would not abolish all punishment. There are cranks who would, and there are cranks scarcely less cranky who would, without abolishing the name, abolish the thing by taking all or almost all the sting out of it. If they had their way, the punishment of a stupid, brutal, cruel, inept, disgraceful, vindictive, useless, mischievous, scandalous crime, to borrow a few of Mr. Salt's epithets, would be to place the criminal in quarters far more luxurious than he had known before the crime was committed, and to pamper him with all kinds of indulgences and pleasures. This war has killed many good men: it has killed a few silly fads; it has knocked wisdom into a few perverse heads: but it has been less fatal to the fads than to the cranks who hold them. Flogging is torture: granted. That is not the question at issue. The real questions at issue are whether it is better to punish by a torture that is brief, cheap, easily inflicted, and looked upon by its victims with horror and dread, than to punish by a torture that is prolonged for years, that is very expensive, that employs a large staff of persons in unproductive work, and that its victims dread very little. In some cases, as, for instance, for offences committed by soldiers in the field, imprisonment is impracticable. Of course the cranks who would abolish all punishment would allow no alternative; but the soldiers whose lives have been imperilled by a sentry sleeping at his post would take another view; and if some corporal punishment were not inflicted by their officers, they would take the matter in hand themselves, and the culprit would certainly not have reason to rejoice at the intervention. Mr. Salt repeats the silly catchword that the criminal is the product of society, and that the best way in which society can mark its indignation at evil deeds is by reforming itself. The catchword is utterly meaningless, and it is eagerly caught up by the criminals themselves as an excuse for making no attempt at reform. It is therefore as pernicious as it is silly.

**Surgical Contributions.** By Rutherford Morison, M.B., F.R.C.S. Edin., F.R.C.S. Eng. (Bristol: John Wright and Sons, Ltd. 1916. Two vols. Royal 8vo. Pp. 427 and 953. Illustrated. Price 45s. net.)

These two handsome volumes contain the collected works of Mr. Rutherford Morison, Professor of Surgery in the Durham University. The first volume deals with general surgery, the second with abdominal surgery, and both are introduced by a eulogistic preface from the pen of Dr. D'Oyley Grange, of Harrogate, who has acted as editor. It is rarely that a busy surgeon is able to publish a complete record of what he has contributed to the press over a period of thirty-seven years, but, with the help of Dr. D'Oyley Grange, Professor Morison has succeeded in doing so. The two volumes not only contain the various papers, cases, and reviews as they were written, but they are rendered the more valuable by the notes which Professor Morison has added, sometimes justifying his opinions, and sometimes pointing out where he was mistaken.

Professor Morison is well known as one of the great teachers of surgery in the North of England who has done much to raise surgery to its present eminence in that part of the country both by his practice and precept. The papers are not only valuable in themselves, but they are made interesting by the style and manner in which they are presented to the reader. The whole series of lectures, notes of cases, and observations show how steadily surgery has progressed from the time when extension was a new principle in the treatment of acute joint disease and when an abdominal operation was a rarity, to the time when aseptic surgery had almost replaced antisepsis and when it could be said that it was possible to be too zealous in making abdominal explorations.

The teaching throughout is clear and precise. Anyone who reads through the volumes, and more especially the second one, which treats of abdominal surgery, will have gained a very sound knowledge of the most valuable kind—that which is derived from personal experience. There is necessarily some amount of repetition in a series of collected essays written at divers times and for varying audiences, but these repetitions add to rather than detract from the value of the books. As a minor point of criticism it would have been better if the headlines on each page had dealt with the subject-matter of the essay. A repetition of the same heading throughout makes it difficult to find any individual article without referring to the indices.

